# 564 The Impact of COVID-19 on the Provision of Pediatric Burn Care

**DOI:** 10.1093/jbcr/irac012.192

**Published:** 2022-03-23

**Authors:** Hawwa Chakera, Jennifer Zuccaro, Charis Kelly, Joel Fish

**Affiliations:** University of Toronto, Toronto, Ontario; SickKids, Toronto, Ontario; SickKids, Toronto, Ontario; Hospital for Sick Children, Toronto, Ontario

## Abstract

**Introduction:**

The WHO declared the outbreak of COVID-19 a pandemic in the spring of 2020 which led to widespread restrictions on daily life activities as people were instructed to isolate at home. Given that 75 – 85% of pediatric burns occur in the home, it is likely that these measures had an impact on pediatric burn care. Thus, the aim of this study was to investigate the impact of the COVID-19 pandemic on the provision of pediatric burn care at an American Burn Association-verified pediatric burn center.

**Methods:**

Data was retrospectively extracted from all new burn patients aged 0-18 years during a pre-pandemic period (April 2019 – August 2019) and a pandemic period (April 2020 – August 2020). Continuous data was examined using 2 tailed t-tests (p < 0.05), while non-continuous data was examined using Pearson chi-squared tests (p < 0.05). These analyses were used to analyze burn demographics and examine changes in the delivery of acute and follow-up burn care before and during the pandemic.

**Results:**

During the pre-pandemic period, 213 new burns were identified, compared to 172 new burns during the pandemic period. No clinically significant changes were observed in patient age at presentation (p = 0.54), total body surface area of burn (p = 0.85), and time to presentation following the injury (p = 0.24). Interestingly, a significant increase in friction burns (p = 0.023) was observed, which mainly consisted of treadmill burns. During the pandemic, burn operating room utilization remained high and represented approximately 25% of the hospital's total surgical capacity. In addition, there were no significant changes to inpatient and outpatient encounters (p = 0.56 and p = 1.00) between the two periods thereby highlighting the need for these essential services during the pandemic.

**Conclusions:**

Burn-related service needs remained consistent across the pre-pandemic and pandemic cohorts as demonstrated by the number of new burns as well as the continued provision of burn care. Overall, no clinically significant changes to patient demographics, aside from the increase in friction burns, were observed. Furthermore, the ability to provide all aspects of pediatric burn care at this tertiary pediatric hospital remained consistent across the pre-pandemic and pandemic cohorts. Although this study presents data from the first five months on the pandemic, further analysis of the entire year will be carried out in order to identify additional trends.

